# Delay Discounting as an Index of Sustainable Behavior: Devaluation of Future Air Quality and Implications for Public Health

**DOI:** 10.3390/ijerph14090997

**Published:** 2017-09-01

**Authors:** Meredith S. Berry, Norma P. Nickerson, Amy L. Odum

**Affiliations:** 1 Department of Psychiatry and Behavioral Sciences, Johns Hopkins University School of Medicine, Baltimore, MD 21224, USA; 2 Department of Society and Conservation, University of Montana, Missoula, MT 59801, USA; norma.nickerson@umontana.edu; 3 Department of Psychology, Utah State University, Logan, UT 84322, USA; amy.odum@usu.edu

**Keywords:** air quality, respiratory health, public health, delay discounting, decision-making, intertemporal choice, sustainability, behavioral economics, environment

## Abstract

Poor air quality and resulting annual deaths represent significant public health concerns. Recently, rapid delay discounting (the devaluation of future outcomes) of air quality has been considered a potential barrier for engaging in long term, sustainable behaviors that might help to reduce emissions (e.g., reducing private car use, societal support for clean air initiatives). Delay discounting has been shown to be predictive of real world behavior outside of laboratory settings, and therefore may offer an important framework beyond traditional variables thought to measure sustainable behavior such as importance of an environmental issue, or environmental attitudes/values, although more research is needed in this area. We examined relations between discounting of air quality, respiratory health, and monetary gains and losses. We also examined, relations between discounting and self-reported importance of air quality and respiratory health, and nature relatedness. Results showed rapid delay discounting of all outcomes across the time frames assessed, and significant positive correlations between delay discounting of air quality, respiratory health, and monetary outcomes. Steeper discounting of monetary outcomes relative to air quality and respiratory health outcomes was observed in the context of gains; however, no differences in discounting were observed across losses of monetary, air quality, and respiratory health. Replicating the sign effect, monetary outcomes were discounted more steeply than monetary losses. Importance of air quality, respiratory health and nature relatedness were significantly and positively correlated with one another, but not with degree of delay discounting of any outcome, demonstrating the need for more comprehensive measures that predict pro-environmental behaviors that might benefit individuals and public health over time. These results add to our understanding of decision-making, and demonstrate alarming rates of delay discounting of air quality and health. These results implicate a major public health concern and potential barriers to individual and societal behavior that reduce pollution and emissions for conservation of clean air.

## 1. Introduction

### 1.1. Effects of Poor Air Quality on Public Health

Nearly seven million premature deaths occur worldwide each year as a result of poor air quality [1,2]. The detrimental effects of poor air quality on public health are well documented. Critical questions remain, however, regarding the psychological factors driving individual and societal-level decision-making that results in continued pollution and persisting poor air quality (e.g., continued private car use, lack of restrictions on factory emissions). Understanding factors contributing to persisting pollution resulting from human behavior could facilitate reduced pollution and improved air quality, and ultimately a reduction in the overwhelming morbidity and mortality associated with poor air quality.

### 1.2. Delay Discounting, Poor Air Quality, and Impacts on Public Health

Delay discounting refers to the decrease in value of an outcome with delay to receiving the outcome [3]. Degree (also referred to as “rate” in economics) of delay discounting is related to numerous maladaptive behaviors including drug and alcohol use and abuse [4,5], pathological gambling [6], and high percent body fat [7]. Chabris and colleagues [8] concluded that even with a brief laboratory task, degree of delay discounting might be the single best predictor of real-world human behavior currently available.

Although decades of research have been devoted to relations between delay discounting and individual health related behaviors (e.g., smoking, drug use), only relatively recently have economists (and even less so psychologists) begun to experimentally isolate degree of delay discounting as it relates to sustainable natural resource practices and related public health concerns [9]. A central focus of research has emerged to identify the extent to which individual and societal delay discounting may hold implications for pressing environmental issues such as air quality, and intimately related public health concerns such as respiratory health, lung cancer, and 7 million annual premature deaths worldwide due to poor air quality [1,10,11]. Little is known about the relation between air quality discounting and discounting of other commodities (e.g., respiratory health or money), or relations between traditional measures of pro-environmental behavior and delay discounting of targeted air quality outcomes. If public policies designed to reduce emissions are to be effective, they should reflect reasonable support from society in general, and particularly how society actually discounts air quality and related respiratory health.

To directly address the future of air quality and current emissions, some economists and policy makers have stressed adopting a degree of “zero discounting” (or the lowest possible) to promote clean air quality in the future [12]. Zero delay discounting implies that the future cannot be discounted relative to the present. In other words, to conserve air quality in the future, citizens and policy makers must act in ways that drastically reduce negative anthropogenic influences on air quality *immediately* (e.g., substantially reduce emissions now from industry/factory/private car use). “Zero discounting” however, does not reflect how individuals or the public actually discount future air quality outcomes, at least at short time frames that have been tested.

Rather, the value of an outcome declines hyperbolically, with sharper decreases in value occurring at shorter delays relative to longer delays [13]. Therefore, if a resource manager is unable to commit to a policy, following the logic of hyperbolic discounting, the temptation to reevaluate the policy at a future date could lead to more impulsive decisions of resource consumption than originally planned [14]. Research on hyperbolic discounting of air quality for public health decision-making, as in the present study, is therefore needed (see also [15,16,17,18,19,20], for examples of delay discounting applied to other environmental issues). Additionally, choice procedures designed specifically to target delay to environmentally relevant outcomes may measure different aspects of pro-environmental behavior propensity. Indeed, valuable evidence shows that concern for an environmental issue does not necessarily translate into behavior to resolve the issue. In a study conducted by Kaplan et al. [15]; see also [16], participants rated their concern for environmental outcomes using a visual analog scale (VAS), as well as what percentage of their time they would be willing to devote to solve the issue, across delay, social, and probability discounting scenarios. Participants consistently rated concern for the environmental outcome significantly higher than the time they would spend solving the issue, illustrating across varying scenarios and types of environmental discounting that concern for an environmental issue does not necessarily match action or behavior dedicated to remediating the issue.

In several studies Hardisty and Weber [21] assessed participant choice of hypothetical financial, air quality, and a generic health gains and losses scenario across two delays. The authors found that a hyperbolic discounting model described the data for all outcomes. Within-subject analyses revealed that individuals who discounted gains steeply in one realm (e.g., air quality, health) also discounted gains steeply in other realms (e.g., monetary) and vice versa. Replicating previous research gains were discounted more steeply than losses across all commodities (i.e., the sign effect) [22]. Evidence from Hardisty and Weber [21] demonstrates that individuals who discount future outcomes steeply tend to respond in this way across many commodities for time scales of 1 and 10 years. Berry and colleagues [23] expanded upon the work of Hardisty and Weber [21] by showing that with longer time scales of up to 75 years, strong positive correlations were observed across commodities, and that people who discount future air quality outcomes steeply also discount future monetary outcomes steeply.

Different ideas, however, have been drawn by Gattig and Hendrickx ([24], see also [15,21]) who concluded that based on a review of the literature, choices for environmental outcomes (risks in particular) across time are fundamentally different from choices for financial outcomes. According to Gattig and Hendrickx [24], differences in choices between environmental and monetary outcomes may be a result of increased ethical concerns and emotion associated with environmental choices. More within-subject research using the same methodology (rather than conclusions drawn from multiple studies using different methodologies) is needed to draw firm conclusions on relations between decision-making processes associated with delayed air quality, health, and financial outcomes for theoretical and public health policy implications.

### 1.3. The Present Study

At present there is almost no examination of how people discount air quality (or respiratory health or financial outcomes) across long-term time scales that policies are designed for. There is also little research examining the relation between delay discounting to other indices of sustainable behavior. Pollution emitted now (e.g., anthropogenic CO_2_), can remain in the atmosphere for months to thousands of years [25]. For example, the Community Multi-scale Air Quality Model (CMAQ), derived from atmospheric modeling and analysis research within the United States Environmental Protection Agency (EPA), has modeled increasingly complex air pollution issues designed to assist local communities, states, and countries respond to air pollution projections that will directly affect public health. Scientists have used this model in conjunction with a range of low continuous discount rates to understand how individuals and society can respond to these projections within a cost benefit analysis. However, little empirical research is available on how people and society *actually* discount future air quality to guide such modeling scenarios, especially across longer time horizons. As carbon abatement programs produce benefits that may not occur until far into the future, it is critical to understand how individuals discount air quality on a broader time scale than is usually assessed in delay discounting research (e.g., previous delays have only extended to 25 years). Further, no previous research has examined relations of delay discounting of air quality to other metrics thought to assess concern for the environment or sustainable behavior. Aspects of delay discounting, along with other psychological barriers of engaging in sustainable behaviors, have been recognized (e.g., competing ideological worldviews, perceived risks of change, see [26]), although remain vastly understudied.

The present study was therefore designed to fill these gaps by: (1) examining correlations between degree of delay discounting across air quality gains and losses, a novel respiratory health gain and loss task tied directly to air quality, as well as monetary gains and losses (for comparison to previous discounting experiments) across long time horizons of up to 75 years; (2) testing for differences in discounting across commodities (e.g., air quality, respiratory health, and monetary) and across sign (i.e., valence; gains versus losses) and (3) examining relations between degree of delay discounting and nature relatedness (thought to measure how individuals relate to nature and the propensity to engage in pro environmental/sustainable behavior). This information will be directly relevant as initial information to build upon for policy makers, agencies, and health officials at local, state and national levels involved in modeling future pollution and emissions scenarios related to air quality and public health (e.g., Missoula County Environment Health Division: Air Pollution Control Board, Bay Area Air Quality Management). Such tests will facilitate closing the gap between policy and actual citizen behavior for more realistic and thereby successful goals in emissions reduction and improved public health resulting from cleaner air.

## 2. Materials and Methods

### 2.1. Participants

Individuals registered as Workers on Amazon Mechanical Turk were recruited to serve as participants in the present study. Workers were eligible to view and participate in the study if they: (1) resided in the United States and (2) if they were 18 years of age or older. The brief study description noted that participants would be asked to make decisions about money, the environment, and their health. Participants who completed the survey were compensated $1.50. The University of Montana Institutional Review Board approved all experimental procedures (IRB #91-15).

### 2.2. Materials and Procedure

Participants completed the experiment at their leisure on their own device in a location of their choosing. Before completing any measures, participants were asked to make sure they could complete the tasks undisturbed and focus on the choices presented. Participants completed ten practice trials with money as the outcome to familiarize with time-value tradeoffs. Following practice trials, participants completed three delay-discounting tasks (described in detail below)—a hypothetical monetary discounting task, an air quality discounting task, and a respiratory health discounting task. Within each task, both gains and losses were presented, and the order of presentation (i.e., either gains first, or losses first) was randomized across participants. A monetary delay-discounting task was presented first to acquaint participants with the simplest choice scenario. Then, the presentation order of the remaining discounting tasks (the air quality and health discounting tasks) were randomized with the exception of grouping by sign (gain/loss). For example, if the air quality discounting task was presented, both the air quality gain scenario, and the air quality loss scenario, would be completed prior to presentation of the health discounting task [21].

Participants then answered two additional questions using a Likert scale “How important is your respiratory health to you?” How important is your local air quality to you? with (1) representing “Not Important” and (10) representing “Extremely Important.” Participants also answered the yes or no question “Have you experienced fluctuations in local air quality in the past two years?” The 21-item nature relatedness scale was also administered as this is thought to measure a propensity for sustainable behavior [27]. Lastly, participants answered basic demographic questions—sex, ethnicity, age, and years of education. All experimental events and data recording were programmed using Qualtrics^®^.

### 2.3. Delay Discounting Decision-Making Tasks

#### 2.3.1. Monetary Gain Scenario

The purpose of the delay discounting procedures was to determine how participants valued an outcome as it became temporally distant. Participants were instructed that there are no right or wrong answers, and to simply choose the hypothetical outcomes that they would prefer [28,29,30]. In the hypothetical monetary task, participants chose between a small, immediate amount of money and a larger, delayed amount of money. Participants were told to imagine they won a local lottery, and the lottery commission was giving them the choice to receive money now or after a delay. They must decide how much they would like to receive either now or after a delay. The small rewards were presented in a descending fixed sequence order of dollar amounts ($100.00, $99.00, $95.0, $90.00, $85.00, $80.00, $70.00, $60.00, $50.00, $40.00, $30.00, $20.00, $15.00, $10.00, $5.00, and $1.00), and the delayed amount remained constant at $100.00. Dollar amounts were presented in that order at each delay. Each choice screen read “Would you rather have (*amount*) now, or (*amount*) in (*delay*)?” The indifference point (i.e., the point at which the smaller sooner outcome was equal in value to the larger later outcome) for each delay was defined as the last immediate value chosen, replicating a method used extensively in previous discounting experiments e.g., [28,29]. The delays tested were 1 day, 1 month, 1 year, 5 years, and 75 years in that order. Participants were told to imagine that they would be alive and would receive the outcomes they choose for this task as well as in the air quality and respiratory health scenario.

#### 2.3.2. Monetary Loss Scenario

In this scenario, participants made choices about losing money across time. Participants were told they received a parking fine and must pay the money now (a smaller amount) or in the future (a larger amount). The same amounts and similar question wording were used in the monetary loss scenario as in the monetary gain scenario. The identical descending fixed sequence order as well as the same delays tested in monetary gains scenario were used. The indifference point was defined as the first immediate value chosen.

#### 2.3.3. Air Quality Gain and Loss Scenarios

In the air quality task participants were told to imagine the following scenario:

“Image the local county government is considering a temporary change to its emissions policy to study the effects of air quality on human health and local wildlife. The Air Quality Index, which is a metric designated by the United States will be used to measure the amount of five pollutants (particulate matter, carbon monoxide, sulfur dioxide, nitrogen dioxide, and ground level ozone) which are currently in the air in your area. Air Quality Index ratings range from 0 (very good air quality) to 500 (hazardous air quality). The particulate output and pollutants of nearby factories and power plants would be substantially reduced (or increased) by the changes in emissions policies--and these alterations will significantly improve (or worsen) air quality in your area. These tests will occur for a number of days, after which time the air quality index would return to its former level, but policy makers are also considering making the changes at different intervals in the future. There are no right or wrong answers. Please just choose the outcomes you would prefer [20].”

Each choice screen read “Would you rather have [*days of improved/worsened air quality*] now, or (*days of improved/worsened air quality*) in (*delay*)?” To remain consistent with the monetary discounting task, the small air quality outcomes in days were presented in a descending fixed sequence order (100, 99, 95, 90, 85, 80, 70, 60, 50, 40, 30, 20, 15, 10, 5, and 1 day) and the large air quality outcomes remained constant at 100 days (of improved or worsened air quality). The delays tested and indifference points calculated for gains and losses were identical to the monetary scenario described above.

#### 2.3.4. Respiratory Health Gain and Loss Scenarios

In the respiratory health gain task participants were told to imagine the following scenario:

“Imagine you are in poor health. For the last 2 years you have been ill with a condition similar to asthma that you acquired. At times it is difficult to breathe, especially when you engage in activities that you used to enjoy, such as walking or hiking. As a result of this condition, you also more easily acquire colds and other ailments, some of which require hospitalization. Imagine that without treatment you will feel this way for the rest of your life and that you will remain alive during all of the time periods described here”.

“Fortunately, you may choose between two treatments. One treatment will return you to full health for a number of days right now but will not last and cannot be administered more than once. The other treatment would return you to full health for a longer period of time, although could not be administered until a future date. There are no right or wrong answers. Please just choose the outcomes you would prefer.” [21,30,31].

A similar scenario was presented for the respiratory losses scenario, except the participant was asked to imagine they were currently in full health, but due to some experiences with air quality, their doctor identified sometime in the future they would acquire asthmatic symptoms. Each choice screen read “Would you rather have (*weeks of improved/worsened respiratory health*) now, or (*weeks of improved/worsened respiratory health*) in (*delay*)?” Based on prescreen testing [20] established that health improvements lasting around 12 or so weeks would be valued similarly to monetary and air quality scenarios, and therefore this quantity was used in the present experiment, except expressed in days to remain consistent with the other scenarios. The same delays and indifference point calculations were used as in the monetary gain/loss and the air quality gain/loss scenarios.

### 2.4. Data Analysis

#### 2.4.1. Orderliness of Data

Delay discounting data were considered systematic and used if indifference points across any scenario did not increase across consecutive delays by more than 20 percent of the larger later reward. This criterion is based on the expectation of a monotonically decreasing discounting function [32].

#### 2.4.2. Delay Discounting Data

Two methods were used to characterize the remaining data sets: median indifference points along with *k* parameter values, and Area Under the Curve (AUC). Both are described in detail below. Median indifference points and *k* values were assessed (as *k* values are not usually normally distributed [13]), as well as median AUC data. These data analysis methods: (1) were used to assess and compare measures of delay discounting across monetary, air quality and respiratory health scenarios, and (2) offered a measure of the degree to which future outcomes were discounted.

#### 2.4.3. Indifference Points and *k* Values

Indifference points were generated at each delay and the following widely employed hyperboloid model was used to fit the indifference points with nonlinear regression [33]:

(1) $$V=A/{\left(1+kD\right)}^{s}$$

in which *V* is equal to the value of the outcome at the indifference point (or the value at which the immediate and delayed options are of equal value), and *A* is the amount of delayed reward, and *D* is the delay to receipt of reward and *s* is a scalar of delay and/or amount. *k* represents the degree to which the value of the reward decreases with delay. Higher *k* values indicate steeper devaluation of future outcomes. Hyperbola or hyperboloid models have been used to describe discounting of air quality previously [21,23], and facilitate comparison to previous research and description of how well this hyperboloid describes the data. Using *k* values, we also calculated the delay at which the larger later commodity was discounted by 50% (i.e., effective delay analysis; ED 50) [34,35].

#### 2.4.4. Area under the Curve

We also calculated the median AUC and the AUC value for each participant [36,37]. We used the ordinal scaling transformation of AUC as proposed by Borges et al. [37] to evenly weight the indifference points at each delay (as opposed to greater weighting of longer delays) given the longest delay of 75 years used in the present experiment. AUC values fall between 0 and 1, with lower values representing steeper delay discounting. The AUC values were used for all statistical analyses, and were non-normally distributed with the exception of monetary gains (described below). To test differences in delay discounting (as measured by AUC) across sign (gains vs. losses) within domains of money, air quality, and health outcomes, we used Wilcoxon matched-pairs signed rank tests, (as the data were not normally distributed) while controlling for family-wise error rate using a Bonferroni correction. A Friedman test (non-parametric test used for repeated measures) comparing AUC values across monetary, air quality, and health gains was employed. A separate Friedman test was used to compare AUC values across monetary, air quality, and health losses. Where significant differences were observed, post hoc Wilcoxon matched-pairs signed rank tests using a Bonferroni correction were applied. These analyses are similar to other previous studies comparing multiple commodities [21,23] and signs [20]. These analyses were only applied to AUC values as in previous research [23].

A Spearman correlation (used for non-normal distributions) matrix was also generated to better understand the direction and strength of potential relations between the AUC of gains and losses across monetary, environmental and health outcomes. Additionally, correlations between delay discounting of all commodities and importance of air quality and respiratory health, as well as nature relatedness (21-item NR scale) were examined [27]. Spearman correlations between importance of air quality, respiratory health, and nature relatedness were also calculated. Nature relatedness quantifies how connected one is to nature, and is thought to be loosely related to engaging in sustainable behaviors (e.g., recycling). Higher NR scores represent greater connection to nature.

## 3. Results

### 3.1. Orderliness of Data

Of the 205 individuals that participated, data from 54 were not considered further, and omitted listwise (all data including delay discounting data for these participants were omitted) from analysis due to nonsystematic discounting [32] across either the monetary, air quality or health scenarios. The percentage of data eliminated (26.34%) was similar to other discounting studies conducted online [21].

### 3.2. Demographic Characteristics and Importance of Air Quality and Respiratory Health

Table 1 presents demographic characteristics of the sample. Roughly half the sample was female, approximately 79% were Caucasian, and the mean age was 38 years. The majority of participants had completed some college or were college graduates. Overall, individuals reported that local air quality (Mean = 8.72, SD = 1.5; anchors 1 = not important, 10 = extremely important) as well as respiratory health (Mean = 9.07, SD = 1.35) were highly important. Roughly 26% of the sample had experienced fluctuations in local air quality in the past two years.

### 3.3. Delay Discounting of Money, Air Quality, and Health Outcomes

#### 3.3.1. Median Indifference Points and *k* Values

**Figure 1:**
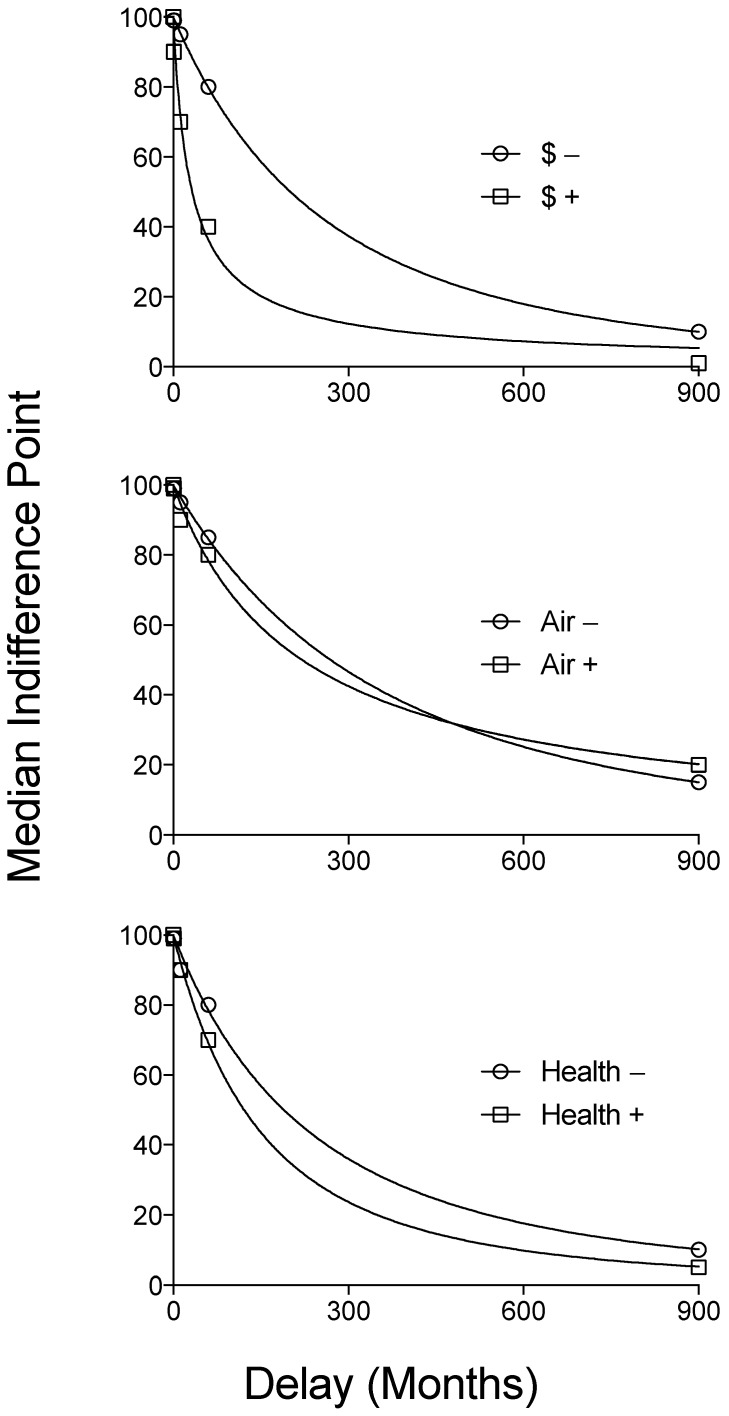
Median indifference points displayed as a function of delay for gains (squares) and losses (circles) across monetary (top panel) air quality (middle panel), and respiratory heath (bottom panel) outcomes.

Figure 1 presents median indifference points across all delays for gains and losses of monetary, air quality, and respiratory health outcomes. Median indifference points decreased as an orderly function of delay across environmental, health and monetary scenarios. Steeper discounting of gains relative to losses was observed for monetary and less so for health outcomes, but no substantial difference in gains and losses for air quality was observed. Median indifference points show that across all outcomes and signs (gains versus losses) the value at 75 years was less than half the value at the next longest delay, 5 years. The median delay at which the value of the larger later amount of money, air quality, and health across both gains and losses decreased by 50% (effective delay, ED50) was less than 15 years for all commodities. Equation (1) provided good fits to the median indifference points for all commodities, and *k* and s values for each commodity are displayed in Table 2. Similar results were obtained when Equation (1) was fit to individual subject data for each commodity, and as expected, resulting *k* values were non-normally distributed for all commodities (assessed by using the Shapiro-Wilk normality test) with the exception of monetary gains.

#### 3.3.2. Area under the Curve

With the exception of monetary gains, all AUC values across monetary losses, as well as air quality and respiratory health gains and losses were non-normally distributed (Shapiro-Wilk normality test), and therefore we used nonparametric statistics. Replicating the sign effect, participants discounted gains more steeply than losses and this effect was especially prominent and statistically significant in the context of monetary outcomes (AUC gains; Median = 0.644, Min = 0.204, Max = 1.00; AUC losses; Median = 0.799, Min = 0.0, Max = 1.00; W_(151)_ = 7036, *p* < 0.0001). Although gains were discounted more steeply than losses across health and air quality outcomes, this pattern was less prominent and not statistically significant in the context of health outcomes (AUC gains; Median = 0.776, Min = 0.028, Max = 1.00; AUC losses; Median = 0.794, Min = 0.0, Max = 1.00; W_(151)_ = 1256, *p* = 0.240), and air quality outcomes (AUC gains; Median = 0.793, Min = 0.241, Max = 1.00; AUC losses; Median = 0.831, Min = 0.0, Max = 1.00; W_(151)_ = 112, *p* = 0.917).

Steeper discounting of monetary outcomes relative to air quality and health outcomes in the context of gains was also observed. A Freidman test (non-parametric test used for repeated measures) comparing AUC values across monetary, air quality and health gains showed significant differences between these commodities (Friedman Statistic _(151)_ = 66.73, *p* < 0.0001). Follow up post hoc Wilcoxon matched-pairs signed rank tests revealed significantly steeper discounting of delayed gains across AUC of monetary outcomes relative to AUC of air quality outcomes (W_(151)_ = 7276, *p* < 0.0001) and AUC of health outcomes (W_(151)_ = 6689, *p* < 0.0001). Differences in discounting of health outcomes relative to air quality outcomes were not observed when controlling for family-wise error rate given the Bonferroni correction (W_(151)_ = −2163, *p* = 0.029). A Freidman test comparing AUC values across monetary, air quality and health losses, showed no significant differences in discounting of money, air quality, or health outcomes (Friedman Statistic _(151)_ = 1.33, *p* = 0.513) and therefore post hoc tests were not used for further examination.

### 3.4. Area under the Curve and Importance of Air Quality, Respiratory Health, and Nature Relatedness

AUC values were used to generate Spearman correlations to assess potential relations across domains (money, air quality, health) and sign (gains, losses), and these results are presented in Table 3. Individuals who discounted monetary gains less steeply, also tended to discount air quality and health outcomes less steeply, as evidenced by significant and strong positive correlations between delay discounting of monetary gains and air quality and health gains, as well as between air quality and health gains. Discounting of monetary losses was also significantly correlated with discounting of air quality and health losses, and air quality and health losses were also significantly and positively correlated. Importance of air quality and respiratory health, and nature relatedness were not significantly correlated with discounting of any outcome, although were significantly and positively correlated with one another (importance of air quality and respiratory health, Spearman correlation as importance of respiratory health was not normally distributed, *r* = 0.743, *p* < 0.001; nature relatedness and importance of respiratory health, Spearman correlation; *r* = 0.367, *p* < 0.001; nature relatedness and importance of air quality, *r* = 0.444, *p* < 0.001).

## 4. Discussion

Several notable results emerged from the present study in which we examined delay discounting of future air quality, respiratory health, and monetary outcomes in the context of gains and losses. First, discounting of one domain (e.g., money) tended to be significantly and positively correlated to discounting of other domains (e.g., air quality, health). Second, gains across money, but not health or air quality were discounted more steeply than losses in the same domains. Third, significantly steeper discounting of monetary gains relative to air quality and health gains, but no differences in discounting of losses across commodities was observed. Finally, importance of air quality, respiratory health, and nature connectedness were significantly and positively correlated with one another, yet not correlated with delay discounting of air quality, respiratory health, or monetary outcomes. Limitations of this experiment and the implications of these results will be discussed in the context of emissions reduction and public health.

Replicating previous research we showed that delay discounting of future air quality, respiratory health and monetary outcomes were significantly and positively correlated with one another [21]. In other words, those who tend to discount the future steeply in the context of monetary outcomes, also tend to discount the future steeply in the context of air quality and respiratory health. The majority of correlations in the present experiment yielded large effect sizes based on Gignac and Szodorai’s [38] empirically extended effect size guidelines (0.30 considered large effect size from meta-analytically analyzed data). This experiment lends additional evidence to the concept of delay discounting as a relatively enduring trait across commodities [39], that also has state-like, malleable characteristics.

Although reasons behind potential differences in degree of delay discounting of money and air quality/environmental outcomes have received attention in environmental literatures, almost no research has been devoted to the potential to *manipulate* greater valuation of environmental outcomes in general, or air quality more specifically. This might be accomplished in the same way as has been shown with money with subsequent influences on real world decision-making (e.g., future episodic thought resulting in less steep discounting of money, and less *ad libitum* energy consumption in obese individuals) [40]. Such research would suggest that the array of techniques used to decrease delay discounting of monetary outcomes (e.g., working memory training, exposure to nature, future episodic thought, and framing effects), might also be used to decrease delay discounting of environmental (and health) outcomes, and would lend evidence to targeting the same underlying processes for greater future valuation across these domains with real-world decision-making implications [40,41,42,43,44,45,46,47,48]. No such studies of this type currently exist, with the exception of a recently published air quality study showing that the same framing effects that reduce discounting of money, also reduce discounting of air quality [26].

Gains were discounted more steeply than losses within monetary outcomes, but not significantly so for health or air quality outcomes. Steeper discounting of gains relative to losses is a well-established finding (the sign effect) [31,49]. The present results replicate this finding in the context of money. Unlike the present study, Hardisty and Weber [21] did show an effect of sign with their air quality discounting task, although they explored discounting across much smaller time windows. It is possible that the lack of the sign effect observed in the context of air quality is due, at least in part, to the very long delay (i.e., 75 years) examined in the present study at which air quality gains or losses simply held little value. Evidence of the sign effect in the context of health and air quality is observed in the predicted direction at shorter delays (e.g., losses discounted less steeply than gains at the 5 year delay; see Figure 1, middle panel). The present findings—in combination with the complexities of actual environmental decision-making that can encompass aspects of gains and losses (e.g., individual versus shared commodities, probabilistic nature of delayed outcomes)—highlight the need for additional research of respiratory health and air quality discounting across longer delays [21]. Such research provides implications for the impact of current decision-making on future improvements and decrements in air quality.

Significantly steeper delay discounting of monetary gains relative to air quality and respiratory health gains were found, although no differences in monetary, air quality, or health losses were observed. This finding, however, must be qualified by the fact that the relative value of each commodity at zero delay was not assessed. Although contingent valuation methods can be used to equate the value of money and environmental outcomes, it should also be noted that some individuals do not believe that environmental commodities should be fungible (i.e., some participants disagree with statements asking whether air quality should be tradable for money) [21], which is why we did not use such valuation methods here. Methodologies employed that equate air quality to money, as well as those that do not will be valuable to understanding the discounting of air quality in the context of broader decision-making. These results should be considered in the context of these limitations. These findings are also in contrast to the findings of Hardisty and Weber [21] in which no differences in discounting were observed across monetary, air quality or health gains. In the present study we presented monetary outcomes first to familiarize participants with the time-value tradeoffs using the simplest scenario possible. Hardisty and Weber [21], on the other hand, randomly presented each discounting commodity to participants. Steeper monetary discounting in the present study could be influenced by order effects.

Importance of air quality, respiratory health, and nature connectedness were significantly and positively correlated with one another. These variables were not, however, significantly correlated with degree of delay discounting for any commodity. This finding aligns with recent conceptualizations that delay discounting may offer a measure of propensity to engage in sustainable behavior beyond that of typical measures of attitudes, values, concern, or connectedness toward the natural environment. Hirsh, and colleagues have argued that delay discounting when presented in an environmental choice paradigm may offer a better evaluation of how a person may actually behave in a choice situation involving delays, which are central in air quality decisions, and which attitude scales do not incorporate [9]. In contrast, popular approaches to pro environmental behavior that emphasize the role of thoughts, concern, values, and attitudes toward the environment fail to access behavior in a choice situation, which are common in day-to-day sustainability scenarios (e.g., drive a private car immediately and increase emissions, or wait for public transportation and reduce emissions; throw the plastic bottle away in the nearby trash can, or travel farther to find the container for recyclables). Indeed, evidence from Kaplan et al. ([15]; see also [16]) shows that concern for an environmental issue does not necessarily translate into behavior to resolve the issue. Choice procedures designed specifically to target delay to improved or degraded air quality, as in the present study, may simply measure different aspects of pro-environmental behavior propensity. Given the strong power of hypothetical choice in delay discounting to predict real decisions [50,51], discounting offers a highly useful framework for approaching environmentally relevant and sustainable behaviors across time [9,14,21,23], particularly in the context of air quality. Future research could investigate the malleability of delay discounting of environmental outcomes and how this might relate to the malleability of real-world, sustainable oriented behaviors that impact air quality.

## 5. Conclusions

Additional research is needed to identify more comprehensive measures that predict pro-environmental behaviors that benefit individuals and public health over time. The present study adds to the growing body of evidence suggesting that humans discount future air quality steeply. Delay discounting across long time frames may be a critical factor for underlying decision-making contributing to sustainable behavior (among other factors [26]) in general and conservation of clean air specifically [23]. By developing and applying techniques designed to decrease the degree to which air quality is devalued in the future as part of a multipronged approach—humans might improve conservation of clean air by engaging in future oriented decision-making that promotes fewer emissions (e.g., riding a bicycle instead of driving a private car, supporting long term air quality emissions reductions, electing officials that promote clean air for future generations). Policy makers may also be more likely to support initiatives that reduce pollution now for benefits in the future. Reducing pollution immediately will be critical to produce both immediate and prospective air quality improvements—as carbon abatement programs frequently produce some benefits that may not occur until far in the future. Substantial reductions in emissions will be required to decrease the devastating morbidity and mortality resulting from poor air quality such as ischemic heart disease, stroke, lung cancer, and premature death [1,2]. Changing human behavior remains critical to emissions reductions. These results may provide initial evidence for characterizing delay discounting of air quality, and have far-reaching and important implications for policy-level action, as well as individual decision-making to promote reduced emissions and improve public health [23].

## Figures and Tables

**Table 1:** Summary of Demographic Characteristics (*n *= 151).

Demographic	Percentage or Mean
% Female	56.29%
% Caucasian	79.47%
Mean Age (SD)	38.3 (12.4)
Grammar School	0.66%
High School/Equivalent	9.93%
Vocational/Technical School	3.97%
Some College	29.14%
College Graduate	39.07%
Master’s Degree	12.58%
Doctoral Degree	1.99%
Professional Degree	2.65%

**Table 2:** R^2^, *k*, and s values for gains and losses of monetary, air, and health outcomes.

Commodity	R^2^	*k*	s
Money +	0.99	0.043	0.798
Money −	0.99	0.001	2.909
Air +	0.99	0.005	0.955
Air −	0.99	0.001	3.223
Health +	0.99	0.003	2.316
Health −	0.99	0.002	2.423

**Table 3:** Spearman AUC Correlation Matrix for Gains and Losses for Monetary, Air, and Health Outcomes.

Commodity	$ +	$ −	Air +	Air −	Health +	Health −
$ +	--					
$ **−**	0.268 **	--				
Air +	0.345 **	0.125	--			
Air −	0.294 **	0.166 *	0.680 **	--		
Health +	0.334 **	0.107	0.622 **	0.468 **	--	
Health −	0.339 **	0.258 **	0.477 **	0.593 **	0.438 **	--

** Significant at the 0.01 level (2-tailed). * Significant at the 0.05 level (2-tailed).
